# Sustained Erythroid Response to Eltrombopag in Low‐Risk Myelodysplastic Neoplasms With Severe Anemia and Thrombocytopenia: A Case Report

**DOI:** 10.1155/crh/1385595

**Published:** 2026-08-26

**Authors:** Ambra Di Veroli, Cinzia De Gregoris, Roberta Laureana, Matteo Caridi, Valentina Panichi, Monica Poscente, Esther Natalie Oliva, Roberto Latagliata

**Affiliations:** ^1^ UOC Hematology, ASL Viterbo, Santa Rosa Hospital, Viterbo, Italy; ^2^ UOC Clinical Diagnostic, ASL Viterbo, Santa Rosa Hospital, Viterbo, Italy; ^3^ UOSD Laboratory of Medical Genetics, ASL Viterbo, Santa Rosa Hospital, Viterbo, Italy; ^4^ Hematology Department, London North West University Healthcare NHS Trust, London, UK

**Keywords:** case report, eltrombopag, erythroid response, myelodysplastic neoplasms, thrombocytopenia

## Abstract

Eltrombopag has been already employed for thrombocytopenia in myelodysplastic neoplasms (MDS), but its role and efficacy in improving other cytopenias are still unknown. In October 2020, a 58‐year‐old low‐risk MDS male with transfusion‐dependent/erythropoietin‐resistant anemia and severe thrombocytopenia (platelets [PLTs]: 19 × 10^9^/L) started eltrombopag in the EQOL‐MDS trial, showing an unexpected increase in Hb values up to 12.0 g/dL at Week 24, with discontinuation of the transfusion requirement. However, PLT levels remained unchanged and, as per protocol, eltrombopag was stopped in May 2021. After eltrombopag discontinuation, Hb levels worsened again with reappearance of transfusion requirement from July 2021. High‐dose erythropoietin and deferasirox (ferritin: 1140 ng/mL) were restarted in September 2021, without efficacy. Thus, in December 2022, eltrombopag was reinitiated on a compassionate basis, and a new progressive increase in Hb (also in PLT values) was observed. After 12 months of 2^nd^ eltrombopag treatment, Hb levels became normal, while PLT count maintained stable values of > 50 × 10^9^/L. At the last follow‐up, the patient was still in complete erythroid response (Hb > 12 g/dL) lasting > 29 months and in complete PLT response (> 100 × 10^9^/L) lasting > 18 months. Eltrombopag could be evaluated as a possible option for treatment of low‐risk MDS with anemia and thrombocytopenia.

## 1. Introduction

Myelodysplastic neoplasms (MDS) are heterogeneous diseases characterized by ineffective hematopoiesis with peripheral cytopenias. Although anemia is the most frequent cytopenia, severe thrombocytopenia (< 30 × 10^9^/L platelets [PLTs]) is reported in about 10% of subjects with low‐risk MDS (LR‐MDS) [1].

Multifactorial mechanisms for the occurrence of thrombocytopenia in MDS have been reported, involving either a differentiation impairment or increased apoptosis of megakaryocytic progenitors, as well as dysregulated thrombopoietin (TPO) signaling and increased PLT destruction through immune or nonimmune mechanisms [1].

Treatment of MDS with severe thrombocytopenia is still limited to PLT transfusions, since no effective drug is currently available for these subjects, thus representing an unmet clinical need [1].

Eltrombopag is an orally available TPO receptor agonist indicated for the treatment of chronic immune thrombocytopenia and severe aplastic anemia (SAA). Beyond the effect on megakaryopoiesis, eltrombopag also showed stimulating effects on hematopoietic stem cells (HSCs), with consistent clinical efficacy in SAA [2].

The Phase‐II randomized study, EQOL‐MDS, has been previously demonstrated, encouraging safety and superiority of eltrombopag in inducing PLT response (PLT‐R) compared to placebo, making this drug a promising approach for the management of thrombocytopenia in LR‐MDS [3].

It is worth noting that in this trial, similar to what was observed in aplastic anemia, a sizable fraction of MDS patients receiving eltrombopag also achieved an erythroid/granulocytic response: a possible mechanism involved in the erythroid effects of eltrombopag could be the activation of multiple kinases by stimulation of the erythropoietin receptor (EPO‐R) in HCS.

Here, we present a case of LR‐MDS with anemia and severe thrombocytopenia treated with eltrombopag, who achieved a significant and durable erythroid response.

## 2. Case Presentation

In December 2019, a 58‐year‐old male was referred to our center for anemia (Hb: 9.9 g/dL) and thrombocytopenia (PLT: 46 × 10^9^/L). Marrow aspirate showed bilineage dysplasia with blast count < 2%, while Perls reaction was negative, and cytogenetic analysis showed a normal karyotype The NGS analysis, made with the 30‐gene panel “Myeloid Solution” by Sophia Genetics, identified a DNMT3A mutation (c.977G > A, p.(Arg326His), R326H) with a VAF of 5.0%, belonging to Class 4 (likely pathogenic) according to ACMG/AMP Consensus Recommendation: no other myeloid driver mutations, including SF3B1, and no JAK/STAT‐pathway gene variants were detected. He was thus diagnosed with MDS with low blasts according to WHO 2022 classification and scored in the low‐risk IPSS‐R category.

After 6 months, he developed severe anemia (Hb: 7.5 g/dL) with a high‐burden red blood cell (RBC) transfusion dependence (2 RBC units/month), severe thrombocytopenia (PLT: 26 × 10^9^/L), and mild leukopenia (WBC: 3.28 × 10^9^/L). Though the baseline level of serum erythropoietin was > 500 mIU/mL, standard treatment with high‐dose erythropoietin alfa (40,000 U twice a week) was started in July 2020 without any benefit.

**FIGURE 1:**
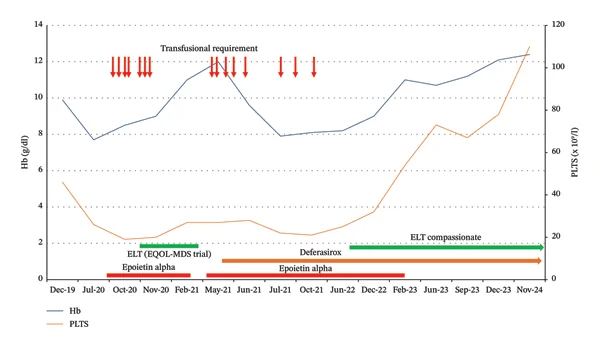
Hemoglobin and platelet values according to treatment in the patient’s follow‐up.

In October 2020, based on severe thrombocytopenia (PLT: 19 × 10^9^/L), the patient was enrolled in the EQOL‐MDS trial in the eltrombopag arm and underwent treatment for 6 months, increasing the dosage of eltrombopag up to a maximum of 300 mg/day. Figure 1 shows the trend of Hb levels and PLT counts from diagnosis and during the entire treatment period.

During the trial, the patient showed an unexpected and progressive increase in Hb values up to 12.0 g/dL at Week 24, requiring reduction and discontinuation of the ongoing erythropoietin treatment. However, PLT levels remained unchanged and, as per protocol rules, eltrombopag was stopped at 24 weeks in May 2021 due to failure to achieve the primary endpoint of the study (increase in PLT count).

After 8 weeks of eltrombopag discontinuation, he developed progressive anemia requiring RBC transfusions from July 2021 (2 RBC units/month). High‐dose erythropoietin alfa was re‐introduced and, due to iron overload (ferritin: 1140 ng/mL), deferasirox treatment was also prescribed in September 2021.

Given the significant erythroid response obtained during eltrombopag, since the drug is not indicated for MDS, an off‐label request to our hospital administration and ethics committee was submitted and approved.

Thus, in December 2022, eltrombopag was restored at the initial dose of 50 mg, with a baseline Hb level of 9 g/dL and PLT count of 32 × 10^9^/L. He required a dose increment to 100 mg after 2 weeks and, by Week 4, a progressive increase in PLT and Hb values were observed. After 4 months, due to a Grade 2 drug‐related increase in transaminases, eltrombopag was reduced to 100 mg alternating with 50 mg/day, with rapid resolution of the toxicity.

After 7 months, erythropoietin treatment was interrupted, and Hb reached normal levels following 12 months of continuous eltrombopag treatment in November 2023. PLT count maintained stable values above 50 × 10^9^/L. Deferasirox treatment was also discontinued in February 2025 due to progressive decrease of ferritin levels to < 300 ng/mL.

At the last follow‐up in April 2026, the patient was in excellent general condition with stable disease in erythroid complete response (Hb: 12.5 g/dL without erythropoietin support) lasting > 29 months and in complete PLT‐R (201 × 10^9^/L) lasting > 18 months: marrow aspirate showed a stable condition with dysplastic features and no evidence of blasts, while DNMT3A mutation was below the limit of detection of the panel at NGS analysis.

## 3. Discussion

Thrombopoietin is the principal regulator of PLT production through binding of the receptor c‐MPL on megakaryocytes, which results in PLT maturation and release. HSC progenitor cells also express c‐MPL, and the addition of recombinant TPO expands the pool of HSC in culture [4].

These observations suggest that stimulation of c‐MPL–signaling pathways may overcome depletion of HSC and support the effect of eltrombopag not only in the case of thrombocytopenia but also in the presence of anemia, as already reported in clinical settings [4].

LR‐MDS with thrombocytopenia remains a clinical challenge. Effective therapies are unavailable, besides PLT transfusions. Erythropoiesis‐stimulating agents and, more recently, luspatercept are valid therapeutic options for patients with LR‐MDS with anemia, but they have no evident effect on thrombopoiesis.

This is the first reported case of LR‐MDS with severe anemia and thrombocytopenia treated with eltrombopag and experiencing resolution of both cytopenias with a durable effect. Noteworthy, there was a possible temporal association between the start of eltrombopag and erythroid response in both treatment phases with eltrombopag, as clearly shown in Figure 1.

An intriguing hypothesis is that the combined stimulation of TPO‐R and EPO‐R could be able to promote activation of multiple kinases within the marrow progenitor compartment, potentially accelerating differentiation and commitment toward both erythroid and megakaryocytic lineages. In both phases of eltrombopag treatment, thus, we cannot exclude a synergistic effect between eltrombopag and EPO in the achievement of erythroid response.

While we observed only an Hb response without PLT increment during the first 24‐week administration of eltrombopag, a complete PLT‐R also occurred during the second eltrombopag treatment. It is worth noting that in this second phase of eltrombopag treatment, we cannot exclude a confounding role of concomitant deferasirox therapy, as erythroid responses were reported in about 20% of LR‐MDS during deferasirox administration. We speculate that the reduction of transfusions and the iron chelation therapy could have concomitantly created a favorable marrow microenvironment. It has been reported that eltrombopag can act as a shuttle molecule, transferring chelated iron to other iron chelators such as deferasirox [5]. This enhances the overall iron chelation process by combining multiple chelators. Biological studies are warranted to confirm this intriguing hypothesis.

Based on this case report and ongoing clinical trials, eltrombopag could be evaluated as a possible option for treatment of LR‐MDS with anemia and thrombocytopenia.

## Funding

This research did not receive any specific grant from funding agencies in the public, commercial, or not‐for‐profit sectors.

## Conflicts of Interest

Roberta Laureana reports honoraria from Bristol‐Myers Squibb, AbbVie, and Novartis and support for attending meetings from AbbVie and Novartis.

Esther Natalie Oliva reports royalties from Ryvu Therapeutics S.A, Halia Therapeutics, and Servier; honoraria from advisory boards from Daiichi Sankyo, Celgene BMS, Sanofi, and Sobi; consultancy fees from Ryvu Therapeutics S.A, Celgene BMS, Daiichi Sankyo, and Janssen; honoraria for presentations from Syros, Sobi, Amgen, Pfizer, Alexion, Novartis, and BeiGene; and support for attending meetings from Celgene BMS, Sanofi, Daiichi Sankyo.

The remaining authors declare no conflicts of interest.

## Supporting Information

Additional supporting information can be found online in the Supporting Information section.

## Supporting information


**Supporting Information** CARE report.

## Data Availability

The data that support the findings of this study are available from the corresponding author upon reasonable request.
