# Supplementary material for: Sustained Erythroid Response to Eltrombopag in Low‐Risk Myelodysplastic Neoplasms With Severe Anemia and Thrombocytopenia: A Case Report
Source: Case Rep Hematol. 2026 Aug 26;2026:1385595. doi: 10.1155/crh/1385595 (PMC13507826; doi:10.1155/crh/1385595)
Supplement: Supplementary file 1 [file CRH-2026-1385595-s001.pdf]

CARE case report guidelines

CARE Checklist of information to include when writing a case report

Topic	Item	Checklist item description	Reported on Line
Title	1	The diagnosis or intervention of primary focus followed by the words "case report"	Lines 1-2
Key Words	2	2 to 5 key words that identify diagnoses or interventions in this case report, including "case report"	Lines 52-53
Abstract (no references)	3a	Introduction: What is unique about this case and what does it add to the scientific literature?	Lines 34-36
	3b	Main symptoms and/or important clinical findings	Lines 36-38
	3c	The main diagnoses, therapeutic interventions, and outcomes	Lines 38-48
	3d	Conclusion—What is the main "take-away" lesson(s) from this case?	Lines 48-50
Introduction	4	One or two paragraphs summarizing why this case is unique (**may include references**)	Lines 54-77
Patient Information	5a	De-identified patient specific information.	Lines 78-80
	5b	Primary concerns and symptoms of the patient.	Lines 80-83
	5c	Medical, family, and psycho-social history including relevant genetic information	Not Relevant
	5d	Relevant past interventions with outcomes	Lines 84-88
Clinical Findings	6	Describe significant physical examination (PE) and important clinical findings.	Lines 85-93
Timeline	7	Historical and current information from this episode of care organized as a timeline	Figure 1
Diagnostic Assessment	8a	Diagnostic testing (such as PE, laboratory testing, imaging, surveys).	Lines 78-83
	8b	Diagnostic challenges (such as access to testing, financial, or cultural)	Not Relevant
	8c	Diagnosis (including other diagnoses considered)	Lines 81-83
	8d	Prognosis (such as staging in oncology) where applicable	Not applicable
Therapeutic Intervention	9a	Types of therapeutic intervention (such as pharmacologic, surgical, preventive, self-care)	Lines 88-108
	9b	Administration of therapeutic intervention (such as dosage, strength, duration)	Lines 106-108
	9c	Changes in therapeutic intervention (with rationale)	Lines 103-108
Follow-up and Outcomes	10a	Clinician and patient-assessed outcomes (if available)	Not Assessed
	10b	Important follow-up diagnostic and other test results	Lines 111-113
	10c	Intervention adherence and tolerability (How was this assessed?)	Not Reported
	10d	Adverse and unanticipated events	Lines 108-110
Discussion	11a	A scientific discussion of the strengths AND limitations associated with this case report	Lines 117-141
	11b	Discussion of the relevant medical literature **with references**.	Lines 117-124
	11c	The scientific rationale for any conclusions (including assessment of possible causes)	Lines 136-141
	11d	The primary "take-away" lessons of this case report (without references) in a one paragraph conclusion	Lines 142-144
Patient Perspective	12	The patient should share their perspective in one to two paragraphs on the treatment(s) they received	Not reported
Informed Consent	13	Did the patient give informed consent? Please provide if requested	Yes ☒ No ☐
